# The impact of temperature and skeletal muscle oxygen saturation on 40 km cycling time trial performance of male cyclists

**DOI:** 10.14814/phy2.70434

**Published:** 2025-07-03

**Authors:** S. H. Faulkner, P. Jobling, N. Wilson, R. James, E. Martin, K. Griggs

**Affiliations:** ^1^ Department of Engineering, SPEED Laboratory, School of Science and Technology Nottingham Trent University Nottingham UK; ^2^ Great Britain Cycling Team National Cycling Centre Manchester UK; ^3^ Department of Sports and Exercise Science, School of Science and Technology Nottingham Trent University Nottingham UK; ^4^ Leicester Lifestyle and Health Research Group, Diabetes Research Centre University of Leicester Leicester UK

**Keywords:** blood flow, cycling, pacing strategy, thermoregulation, tissue oxygenation

## Abstract

Endurance performance declines in non‐heat‐acclimated athletes in warm conditions. Early studies did not use relevant wind speeds, increasing heat storage, and performance decline. Furthermore, deep hip flexion postures adopted in time‐trials (TTs) may limit skeletal muscle oxygenation (SmO_2_), compounding problems associated with heat storage in warm‐hot environments. The aim of this study was to employ an ecologically valid method of simulating TTs, using wind speeds closely replicating those in the real world (~40 km h^−1^). A secondary aim was to investigate how SmO_2_ was impacted by environmental conditions. Ten well‐trained cyclists volunteered for the study. They completed 3 simulated 40 km TT's in 10, 20, and 30 and 50% relative humidity. TT time was slower in 30°C (3666 ± 172 s) versus 10°C (3531 ± 144 s; *p* = 0.0029) and 20°C (3528 ± 160 s; *p* = 0.0033). Mean *T*_sk_ was highest in 30°C (34.4 ± 0.1°C) versus 10°C (25.4 ± 1.5°C) and 20°C (30.6 ± 0.3°C; all *p* < 0.0001). Larger ∆SmO_2_ was evident at 10°C (−1.3 ± 0.7%) and 20°C (−1.2 ± 0.7%) versus 30°C (0.4 ± 0.7%; both *p* < 0.0001). ∆SmO_2_ was correlated to ∆heart rate (*r* = −0.556, *p* < 0.0001) and power (*r* = −0.425, *p* < 0.0001). These data show that simulated TT performance is impaired >20°C.

## 1 INTRODUCTION

Endurance exercise performance progressively deteriorates as the surrounding ambient temperature increases (Tatterson et al., 2000; Tucker et al., 2006), which is exacerbated when combined with increasing humidity (Maughan et al., 2012) and solar radiation (Otani et al., 2019). There is a strong link between increases in thermoregulatory strain, due to elevations in both metabolic and ambient heat, and impaired endurance performance. In the seminal study by Galloway and Maughan, it was shown that endurance capacity is reduced in ambient temperatures more than 11°C (Galloway & Maughan, 1997). However, this study is somewhat limited owing to an air speed of only 0.7 m s^−1^ at a fixed workload, which does not reflect the air speeds experienced by a competitive cyclist who will maintain speeds of ≥40 km h^−1^ with an air speed of ~11.1 m s^−1^. Such a discrepancy in the air velocity a rider is exposed to will limit the capacity for heat loss via evaporative and convective cooling and increase the rate at which heat storage occurs (Parsons, 2014). Practically, this would more than double the maximum evaporative requirement for a 70 kg rider riding at 280 W to achieve heat balance, with the required sweat rate increasing from 1.1 to 2.1 L h^−1^. Furthermore, there will likely be a negative impact of warmer temperatures on perceptual variables that are known to be important in pacing strategy selection and subsequent exercise performance (Faulkner et al., 2015; Flouris & Schlader, 2015; Schlader et al., 2011; St Clair Gibson et al., 2018). Taken together, it is important to establish a threshold temperature above which endurance performance is negatively impacted using a robust, ecologically valid protocol with appropriate wind speeds and allowing for fluctuations in power output in response to fatigue and altering of pacing strategies. Such information will help in designing strategies to mitigate against performance declines in the heat and provide athletes, coaches, and practitioners with a threshold temperature above which external cooling should be used to maintain exercise performance.

Previously we have shown that endurance performance can be improved via the use of pre‐cooling and suggested a temperature threshold for the effectiveness of pre‐cooling of being >24°C (Faulkner et al., 2015, 2019). In the context of the present study, this suggests that above 24°C there is a significant impact of temperature on performance. Indeed, this view has recently been in some way supported in a retrospective study of field‐derived data from professional cycling (Valenzuela et al., 2022). In this study it was shown that in temperatures above ~25°C, there was a decline in mean maximal power profiles, which equated to a 9%–18% decline in performance. This indicates that previous lab‐based studies, and the conditions in which they were conducted, do not meet the same environmental conditions experienced in the field, primarily due to a lack of airflow and therefore convective cooling capacity.

Skeletal muscle oxygen saturation (SmO_2_) is a critical component of local oxygen delivery to working muscles, with greater saturation reflecting increased binding of oxygen to hemoglobin. Redistribution of blood flow to the skin in order to facilitate heat loss occurs during exercise in the heat and will reduce skeletal muscle blood flow and potentially SmO_2_, thereby limiting O_2_ availability and impairing performance. Although global measures of rating of perceived exertion (RPE) and heart rate (HR) increase linearly with power output, they do not provide any insight into the mechanistic underpinnings of pacing regulation and how this may differ in a range of ambient temperatures. Previously, SmO_2_ has been shown to be inversely related to power output during a 20 km time trial (Jaime et al., 2019), and that matching O_2_ delivery to O_2_ uptake becomes progressively challenged during exercise in the severe and extreme intensity domains (Poole et al., 1988; Poole & Richardson, 1997), which are domains commonly frequented during cycling time trials. These factors are likely exacerbated by the postural impact of time trial cycling, where riders typically adopt positions with significant hip hyperflexion, forcing the iliac artery into an acute angle, which impairs lower limb blood flow (Lim et al., 2009). Coupling high‐intensity exercise with extreme environmental temperatures likely results in a greater haemodynamic challenge, owing to an increase in skin blood flow, compared to more temperate environments, with the extent to which this may affect performance being unknown.

### 1.1 Aims

The primary aim of the present study was to determine a threshold temperature above which time‐trial performance is hindered utilizing a more ecologically valid, laboratory‐based methodology than has previously been employed. Secondly, we wished to establish the impact of altered ambient temperature on SmO_2_ and how this relates to TT performance in the heat.

### 1.2 Hypotheses

It was hypothesized that time trial performance would be impaired above 20°C and that this would be associated with alterations in psychological/perceptual and physiological parameters which contribute to the regulation of pacing strategy in the heat.

## 2 METHODS

### 2.1 Participants

Ten well‐trained male cyclists volunteered to take part in the present study (Table 1). All participants were equivalent to performance level 3 ($\dot{V}O_{2}$_max_ 55.0–64.9 mL kg min^−1^; Peak power output 320–379 W; training frequency ≥3 time per week and ≥5 h per week (Pauw et al., 2013)). All participants were provided with a food diary and were asked to record their normal diet and replicate this for the 24 h prior to each experimental trial. They were asked to resume their normal training and refrain from heavy exercise and alcohol 24 h before each experimental trial. Each participant undertook their trials at the same time of day to control for circadian and diurnal variations, with each trial being separated by 7–10 days. The present study was approved by the Nottingham Trent University ethics board and was undertaken in accordance with the declaration of Helsinki. Prior to testing, all participants provided full written informed consent.

**TABLE 1:** Participant characteristics, *n* = 10.

Age (years)	28.1 ± 9.8
Height (cm)	180.4 ± 5.7
Body mass (kg)	76.1 ± 12.0
VO_2max_ (mL kg min^−1^)	59.7 ± 11.1
Maximum aerobic power (MAP; W)	356 ± 13
Anaerobic threshold (LT2; W)	270 ± 15
Weekly training volume (h)	10.5 ± 3.2

### 2.2 Study overview

Participants visited the laboratory on 5 separate occasions. The first visit involved a VO_2max_ test on a cycle ergometer (Lode Excalibur Sport, Groningen, The Netherlands). The test comprised of an incremental test starting at a power output of 95 W with 35 W increments every 3 min until volitional fatigue or until cadence could not be maintained >60 rpm. Oxygen uptake ($\dot{V}$ O_2_), CO_2_ production ($\dot{V}$ CO_2_), respiratory exchange ratio (RER) and heart rate were recorded during the last minute of each stage and were averaged across the final 30 s using an online breath‐by breath analyzer (Cortex, Metalyser 3B, Leipzig, Germany). Fingertip blood lactate samples were collected into 20‐μL capillary tubes, at rest and during the last minute of each stage, and analyzed immediately (Biosen; EKF Diagnostics, Cardiff, UK).

The second visit was a familiarization for the 40 km time trial (see below) undertaken on the participant's own road or TT bike placed on a turbo trainer. The third, fourth, and fifth visits involved three experimental trials, where 40 km time trials were undertaken in either 10, 20, or 30°C, all with 50% relative humidity. All conditions were assigned using a balanced crossover randomization design.

### 2.3 Experimental protocol

Prior to each experimental trial, participants completed the Profile of Mood States (POMS, (McNair et al., 1971)) questionnaire and before familiarization and their final trial to identify how fluctuations in mood may have impacted performance. Participants also completed the Pittsburgh Sleep Questionnaire to identify potential confounding effects on performance (Buysse et al., 1989). To allow for measurement of gastro‐intestinal temperature during the experimental trials, an ingestible telemetric temperature pill (CoreTemp, HQ, Ins, Palmetto, FL) was given to participants. Participants were instructed to swallow the pill ~8–10 h prior to the start of the trials. Pill placement and viability was tested upon arrival to the laboratory using the telemetric receiver. Its position was verified with the ingestion of water. Urine osmolality (Atago Pocket Refractometer PAL‐10S, Japan) and nude body mass (Adam Equipment Co. Ltd., Milton Keynes, UK) were also measured upon arrival to the laboratory. Osmolality was measured to determine hydration status, with acceptable osmolality was characterized as <800 mOsm kg H_2_O^−1^. If a participant was subsequently identified as being dehydrated, they were required to consume 500 mL water and have a subsequent urine osmolality within the required range. Eight wireless thermistors (iButton, DS1922, Sunnyvale, CA) were secured to the skin using porous tape (Transpore™ Surgical Tape, 25 mm, 3 M) in eight separate locations and mean T_sk_ calculated accordingly (ISO 9886, 2014). Subsequently mean body temperature was calculated as (Havenith, 2001):

$$T_{b}=\left(0.8\times T_{\mathrm{gi}}\right)+\left(0.2\times \text{mean}\;T_{\mathrm{sk}}\right)$$

A heart rate monitor was used to continuously record heart rate throughout exercise (Polar R400, Polar, Finland). Water bottles were weighed pre‐ and post‐trial to measure fluid intake. Upon entry to the environmental chamber, two Near Infra‐Red Spectroscopy (NIRS) probes to measure deep tissue muscle oxygenation (SmO_2_; Moor Instruments, Devon, UK) were attached to the skin on the mid‐point of the right vastus lateralis, with 30 mm separation to achieve a measurement depth of 1.5 cm below the skin surface.

### 2.4 Time trial

Both the warm‐up, time trial, and cool‐down were all completed on Zwift™ (Zwift Inc., USA) using the Tempus Fugit route. A Wahoo Kickr (Wahoo, Atlanta, GA, USA) and Wahoo Headwind smart fan (Wahoo, Atlanta, GA, USA) were connected to Zwift via ANT+ to enable the Zwift™ interface to control the fluctuation of air speed from the fan as a function of virtual speed of travel of the bike during the time trials. Additional air flow was provided by a bespoke vertical stack of three 20″ fans positioned 2.0 m away from the front of the rider to provide full body convection.

Following a 2‐min rest period on the bike, participants undertook a standardized 17 min warm up. This consisted of 5 min at 50% peak power output (PPO), 5 min at 60% PPO, 5 min at 70% PPO and 2 min at 80% PPO. After 5 min rest, during which time participants could drink ad libitum and stretch/mobilize as they would prior to a TT, the 40 km TT began from a standing start. They were instructed to complete the TT as fast as possible. Participants were allowed to drink water ad libitum throughout the warm‐up, time trial and cool‐down and were provided live feedback on power output and heart rate. Every 5 km of the target distance, lap power output, lap distance, split time, heart rate, and cadence were recorded (Garmin Edge 830, Garmin, USA). *T*_gi_ (eCelscius, BodyCap, France) was continuously recorded and averaged over the last 60s of each 5 km. The NIRS SmO_2_ probes continuously recorded throughout the entire protocol at 40 Hz. Ambient temperature, relative humidity and wet bulb globe temperature and mean air speed (38.9 ± 4.3 km h^−1^ [10.8 ± 1.2 m s^−1^]; Kestral 5400, Kestral Instruments, Boothwyn, Pennsylvania, USA) were recorded in the last minute of each 5 km. Rating of perceived exertion (Borg, 1982), thermal sensation, thermal comfort (ASHRAE, 1997) and perceived skin wetness, using visual analogue scales were recorded in the last 1 km of each 5 km.

### 2.5 Data analysis and statistics

Data analysis was undertaken using Microsoft Excel, GraphPad Prism (Version 10, GraphPad Software, LLC, USA) and MoorsVMS Software (Moor Instruments, UK). All physiological and performance data were sampled over 90s at rest and during the final minute of each of the following TT time periods: end of the warm‐up, end of 5, 10, 15, 20, 25, 30, 40 km, and the end of the self‐paced cool‐down. SmO_2_ data were analyzed with percentage change reported relative to the baseline measurement. Prior to statistical analysis, all data were checked for normality using the Shapiro–Wilk test. Where data violated normal distribution, they were log‐transformed prior to analysis. Finish time, mean power, sweat rate, and mean ∆SmO_2_ were all analyzed using a one‐way repeated measures Analysis of Variance (ANOVA). Split power, split time, *T*_gi_, *T*_sk_, *T*_b_, heart rate, and SmO_2_ data were analyzed using a two‐way repeated measures ANOVA (temperature *x* time). Correlational data were analyzed using Spearman's *r* statistic. For all analysis, an alpha value of *p* < 0.05 was considered to denote a significant difference between variables. All data are presented as mean ± SD.

## 3 RESULTS

There were no differences in POMS scores or in the Pittsburgh Sleep Questionnaire prior to any of the experimental visits (all *p* > 0.5).

### 3.1 Performance data

**FIGURE 1:**
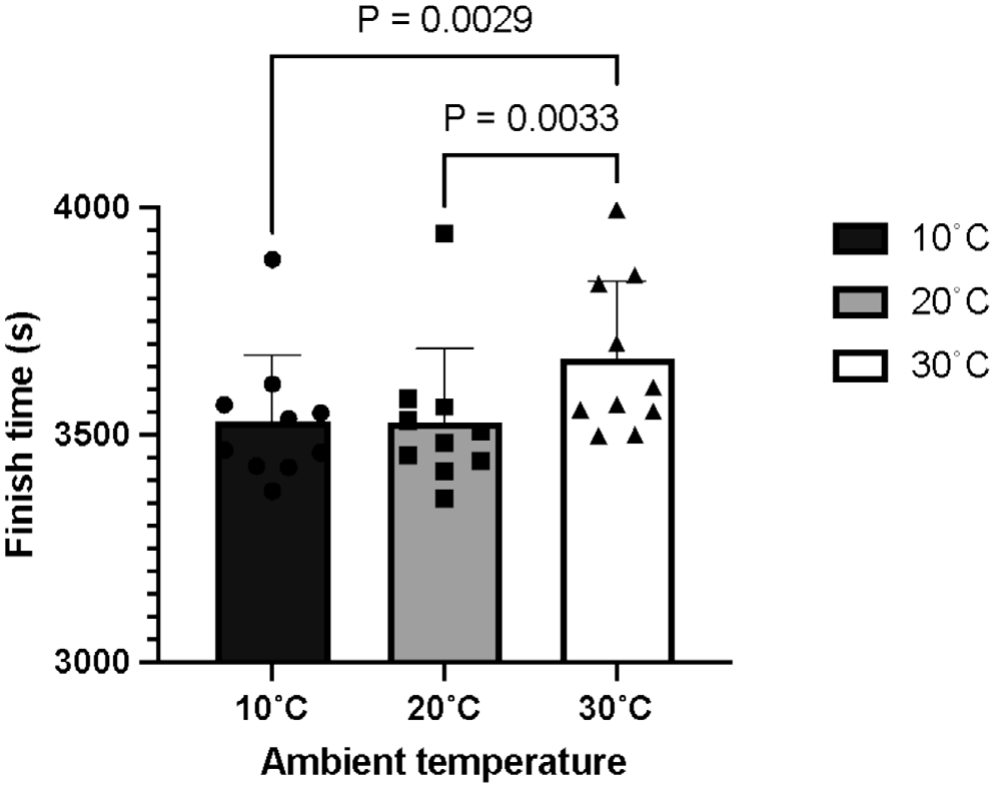
Mean and individual (filled shapes) finish times for a simulated 40 km time trial completed.

There was an effect of temperature on total finish time, with 30°C (3666 ± 172 s) resulting in slower finish times compared to 10°C (3531 ± 144 s; *p* = 0.0029) and 20°C (3528 ± 160 s; *p* < 0.0033, Figure 1). There were no differences in finish time between 10 and 20°C (*p* = 0.9936). This was mirrored in mean power, which was highest for 10°C (281 ± 5 W, *p* = 0.0029) and 20°C (274 ± 10 W, *p* = 0.0033) compared to 30°C (255 ± 10 W). Mean relative power throughout the TT was 3.8 ± 0.1 W kg^−1^ in 10°C, 3.8 ± 0.1 W kg^−1^ in 20°C, and 3.4 ± 0.1 W kg^−1^ in 30°C (*p* < 0.0001 vs. both 10 and 20°C).

### 3.2 Pacing

**FIGURE 2:**
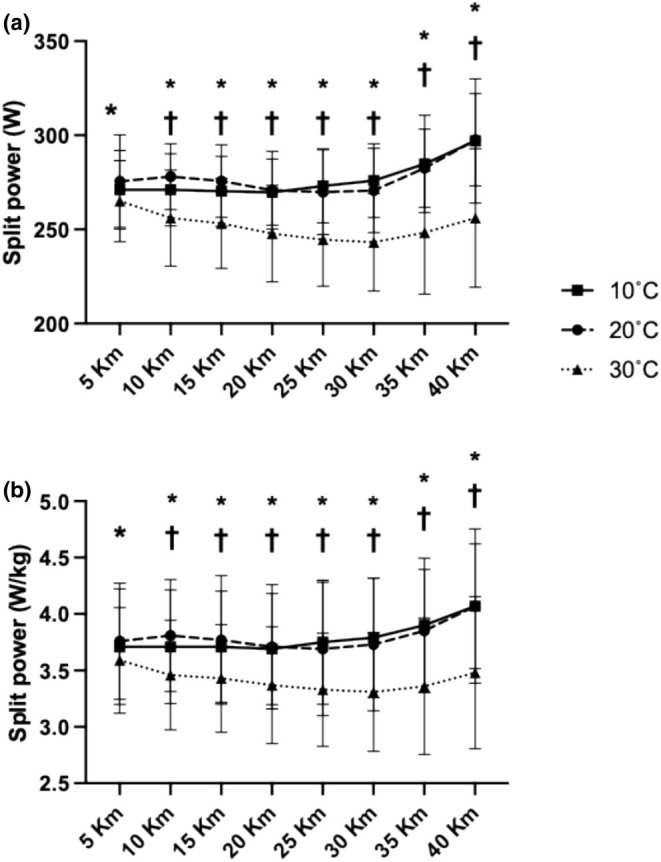
Absolute (a) and relative (b) mean power output for each 5 km split during a simulated 40 km time trial. There were no differences in power between 10 and 20°C at any time point. *Denotes *p* < 0.05 (10 vs. 30°C), †denotes *p* < 0.05 (20 vs. 30°C). *n* = 10.

There were main effects for time (*p* < 0.0001), temperature (*p* < 0.0001) and an interaction effect (*p* < 0.0001) for split time over the course of the 40 km TT. From 5 km, pacing was slower in 30°C compared to both 10°C (*p* < 0.005) and 20°C (*p* < 0.0001), and this lasted throughout the duration of the TT. There were no differences in split time at any stage between 10 and 20°C (Figure 2).

There was a main effect of temperature (*p* < 0.0001) and an interaction effect between distance and temperature (*p* < 0.001) for average power (Figure 2a) and relative average power (Figure 2b). Lower power was evident from 5 km between 30 and 20°C (*p* = 0.0081) and for the duration of the TT thereafter. Lower power was evident between 30 and 10°C from 10 km (*p* < 0.0001) and for the duration of the TT thereafter.

### 3.3 Physiological data

#### 3.3.1 Gastrointestinal temperature (*T*_gi_)

**FIGURE 3:**
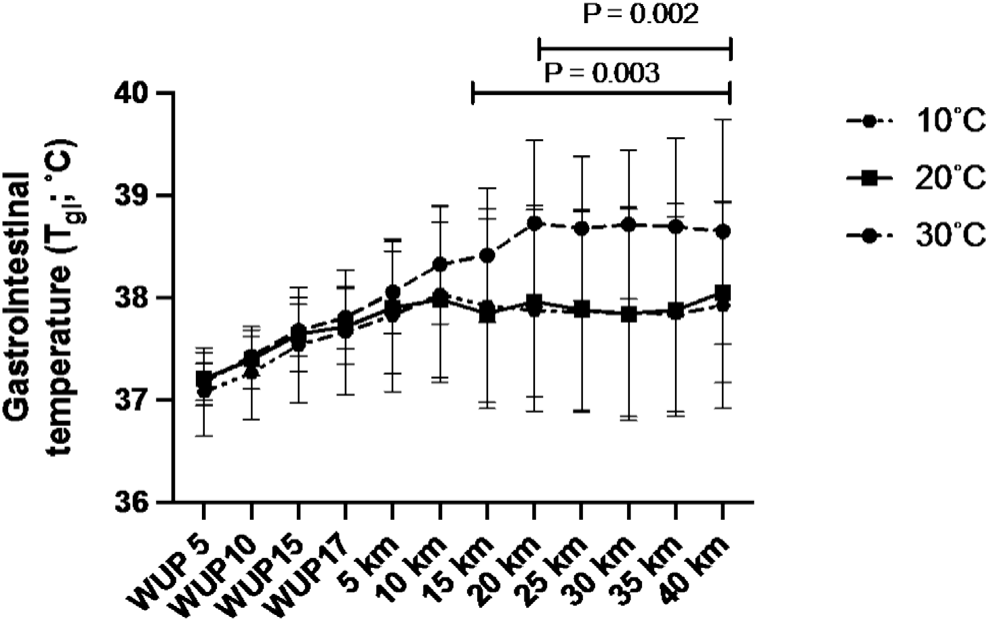
Gastrointestinal temperature throughout the warm up and simulated 40 km time trial. Gastrointestinal temperature was lower in both the 10°C condition and 20°C condition compared to 30°C, with no difference between 10 and 20°C at any timepoint. *n* = 10.

There was a main effect of time (*p* = 0.002), temperature (*p* < 0.0001) and a time *x* temperature interaction for *T*_gi_ (*p* = 0.0488; Figure 3). There were no differences in *T*_gi_ at any stage during the warm‐up or for the first 10 km of the TT. After 15 km, *T*_gi_ was higher for 30°C compared to 20°C (all points *p* < 0.05) and from 20 km compared to 10°C (*p* < 0.0005). These differences remained throughout the rest of the time‐trial.

#### 3.3.2 Mean skin temperature (*T*_sk_)

There were main effects of time (*p* < 0.0001), temperature (*p* < 0.0001) and an interaction effect (*p* < 0.0001) for *T*_sk_. Trial mean *T*_sk_ was highest at 30°C (34.4 ± 0.1°C) compared to 20°C (30.6 ± 0.3°C) and 10°C (25.4 ± 1.5°C; all *p* < 0.0001). Mean *T*_sk_ was different from 5 min of the warm up between all conditions (all *p* < 0.0001) and this effect lasted through the duration of the trial.

#### 3.3.3 Mean body temperature (*T*_b_)

There were main effects of time (*p* < 0.0001), temperature (*p* < 0.0001) and an interaction effect (*p* = 0.001) for Tb. From 5 min of the warm up and throughout the trial, mean *T*_b_ was higher for 20 and 30°C compared to 10°C (both *p* < 0.005). Similarly, mean *T*_b_ was higher for 30°C compared to 20°C from 5 min of the warm up (*p* < 0.0001) and throughout the trial (*p* < 0.005).

#### 3.3.4 Heart rate

There was a main effect of time on heart rate (*p* < 0.0001), but not for temperature (*p* = 0.0763) nor an interaction (*p* = 0.0855). For all points of the warm‐up, heart rate was higher in 30°C compared to 20°C (all *p* < 0.05). Heart rate was higher for 30°C compared to 10°C at 5 min of the warm‐up and the end of the warm‐up only (both *p* < 0.05). There were no differences between 10 and 20°C throughout the warm‐up phase. During the time trial, differences were evident for 10 versus 20°C at 10 km (*p* = 0.0464), 15 km (*p* = 0.040), and 40 km (*p* = 0.034) only. There were no other differences in heart rate.

#### 3.3.5 Sweat rate

There was an effect of condition on total sweat rate estimated using changes in body mass corrected for volumes of fluid consumed (*p* = 0.0021), with sweat rate highest in 30°C (1050 ± 244 mL h^−1^) compared to 10°C (755 ± 175 mL h ^−1^; *p* = 0.0051) and 20°C (755 ± 174 mL h ^−1^; *p* = 0.0059).

#### 3.3.6 Muscle oxygen saturation

**FIGURE 4:**
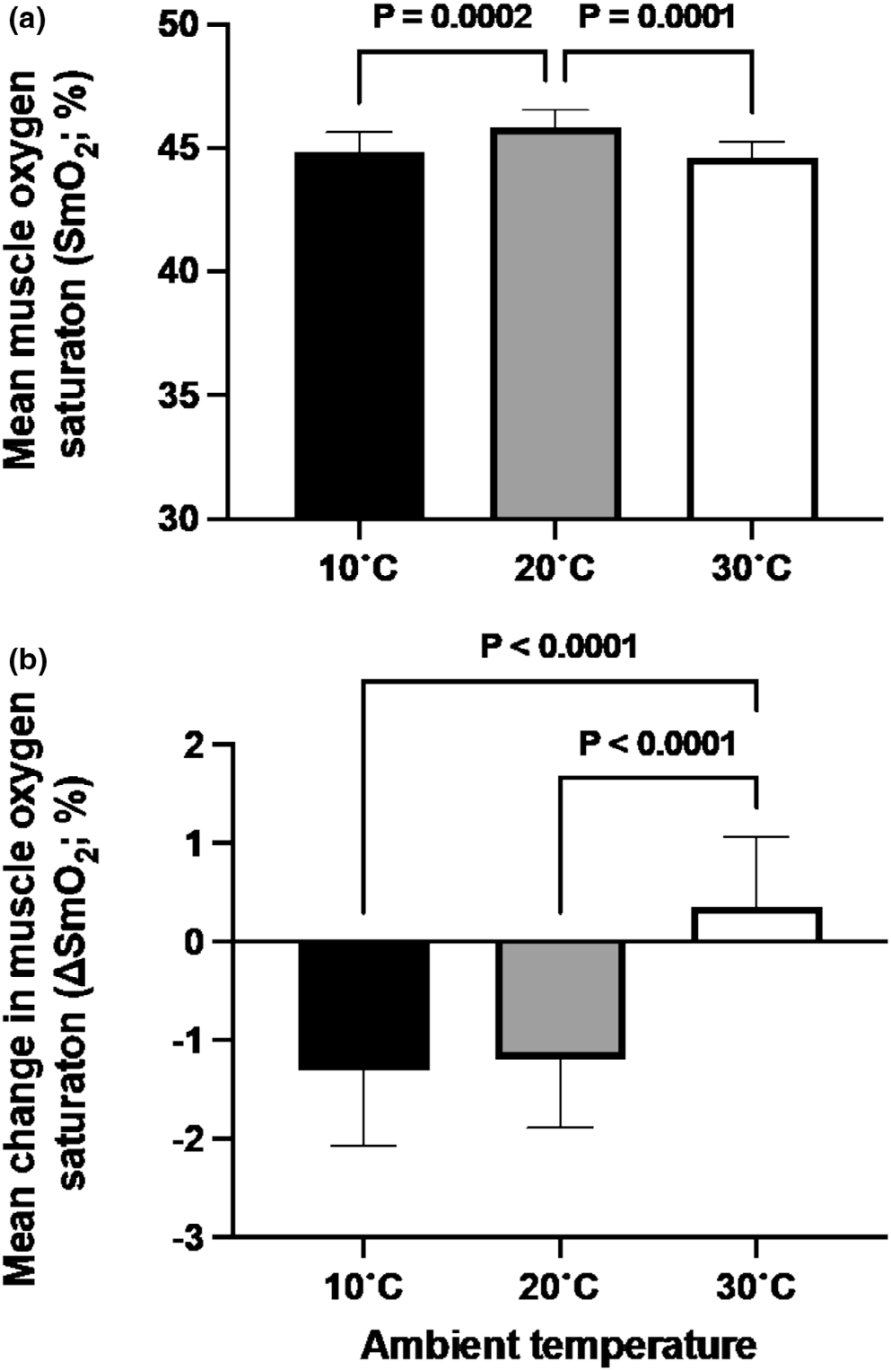
Mean SmO_2_ saturation (a) and change in skeletal muscle oxygen saturation (∆SmO_2_) as a percentage of baseline between conditions (b) during the time trial.

**FIGURE 5:**
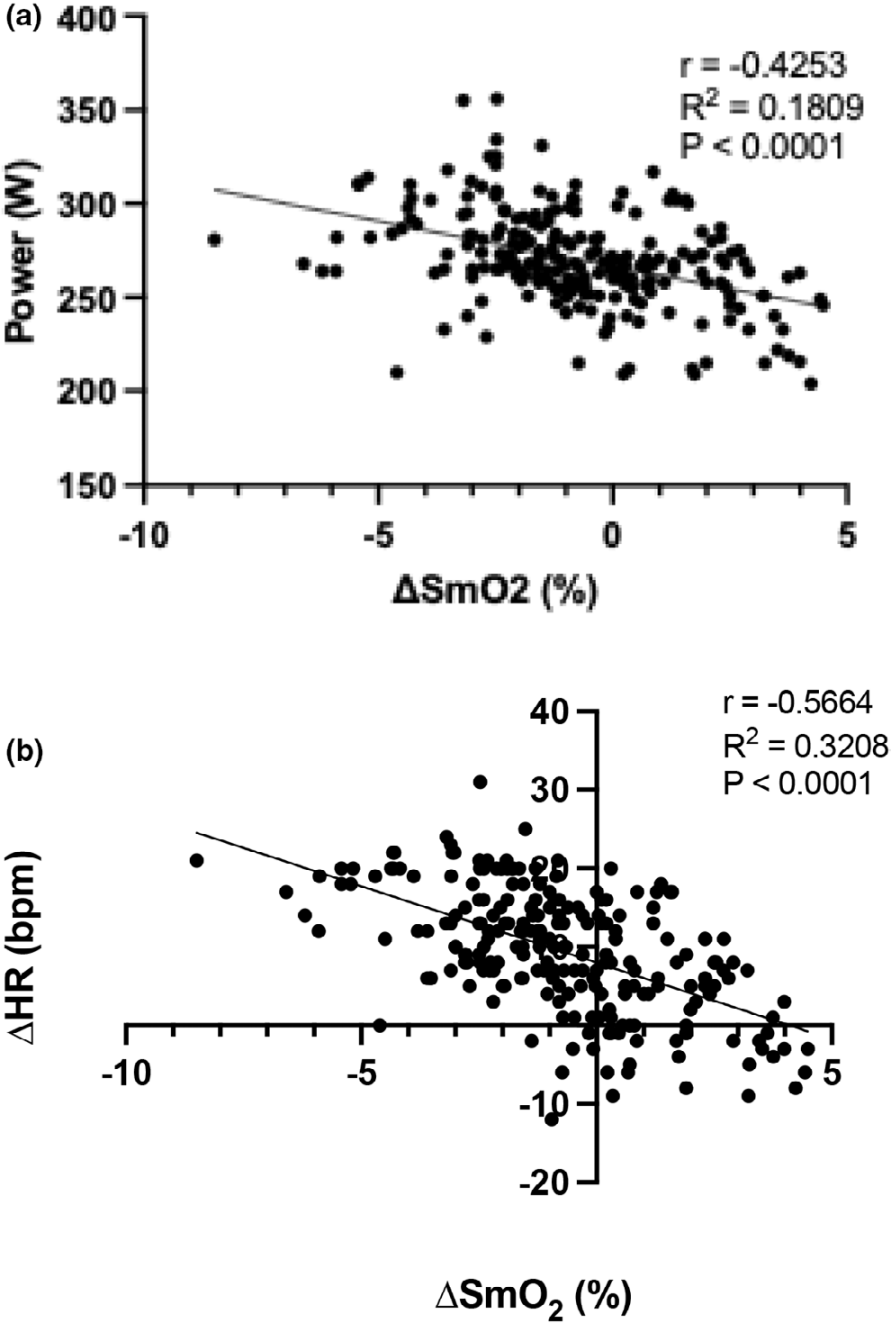
There was an inverse relationship between the change in skeletal muscle oxygen saturation and power output (a) and ∆ heart rate (b). *n* = 10.

There was a main effect of condition on overall mean SmO_2_ during the time trial (*p* = 0.0421). Mean SmO_2_ was lower in 10 versus 20°C (44.9 ± 0.8% vs. 45.9 ± 0.7, *p* = 0.0016, Figure 4a) and higher for 20 versus 30°C (45.9 ± 0.7 vs. 44.6 ± 0.7% *p* = 0.0003). There was a main effect of time on ∆SmO_2_ (*p* < 0.001), but not temperature (*p* = 0.1284). Over the course of the trial, there was an effect of condition on the overall mean ∆SmO_2_ (*p* < 0.0001, Figure 4b), with ∆SmO_2_ being greater for both 10°C (−1.3 ± 0.7%) and 20°C (−1.2 ± 0.7%) versus 30°C (0.4 ± 0.7%; both *p* < 0.0001). ∆SmO_2_ was inversely correlated to mean power (*r* = −0.4253; *p* < 0.0001, Figure 5a) and ∆HR (*r* = −0.5664, *p* < 0.0001, Figure 5b), whereby a greater desaturation resulted in a higher mean power output and change in heart rate throughout the course of the TT.

### 3.4 Perceptual data

#### 3.4.1 RPE

There were main effects of time (*p* < 0.0001) and temperature (*p* = 0.003) on RPE. At 15 min of the warm up (*p* = 0.004) and 17 min of the warm up (*p* = 0.043), RPE was higher for 30°C than 20°C. There were no further differences between conditions during the warm‐up or throughout the TT.

#### 3.4.2 Thermal sensation

There were main effects of time (*p* < 0.0001) and temperature (*p* < 0.0001) on thermal sensation. From 5 min of the warm‐up, thermal sensation was continually higher for 20°C and 30°C, and this persisted for the duration of the warm‐up and TT (all points *p* < 0.005). Differences between 20°C and 30°C were evident for the first 15 min of the warm‐up (all *p* < 0.05) and at 40 km of the TT (*p* < 0.05).

#### 3.4.3 Thermal comfort

There were main effects of time (*p* < 0.001), temperature (*p* < 0.0001) and an interaction effect (*p* < 0.0001) on thermal comfort. Thermal comfort was rated to be more uncomfortable for 30°C through the warm‐up and TT compared to 20°C (all points *p* < 0.001). Only at the end of the warm‐up was a difference evident between 10 and 30°C (*p* < 0.05), which then lasted throughout the TT.

#### 3.4.4 Skin wetness perception

There were main effects of time (*p* < 0.0001), temperature (*p* < 0.0001) and an interaction effect (*p* = 0.0045) on skin wetness perception. From 10 min of the warm up, wetness perception was higher for 30°C compared to 10°C (*p* = 0.011) and 20°C (*p* < 0.048). This continued through to the end of the TT. From 15 min of the warm‐up, wetness perception was greater for 20°C compared to 10°C (*p* < 0.024), which then persisted until the end of the TT.

## 4 DISCUSSION

The main finding of this study was that a performance decrement owing to elevated environmental temperature is evident above 20°C and that this is because of altered pacing from the outset of the time trial. Mechanistically, the altered pacing with lower power output results in a reduction in the desaturation of SmO_2_ at 30°C compared to lower temperatures. This indicates that power output is not limited because of O_2_ delivery within the muscle, but due to an increase in whole body heat stress and its associated impact on pacing modulation. However, it is not possible to conclude whether the change in SmO_2_ occurs because of altered pacing, or that oxygen transport at the muscle is limited during exercise in the heat owing to an increased haemodynamic demand. Consequently, we can accept our hypothesis that time trial performance will be impaired in temperatures above 20°C.

It has previously been demonstrated that exercise capacity in the heat is impaired and that an optimal temperature for endurance performance may be in the region of 11°C (Galloway & Maughan, 1997). However, this study was limited in ecological validity for the context of performance as they employed a test of exercise capacity (e.g., a time to fatigue test) as opposed to a freely paced time trial. Furthermore, the very low air speeds in the Galloway and Maughan study (~0.7 m s^−1^) do not reflect the wind speeds experienced by a cyclist on the road, which likely exceed 12.5 m s^−1^ (45 km h^−1^). This discrepancy is likely a large factor in the difference we report in the present study as the low air speed will severely limit evaporative cooling, which, at high metabolic work rates in warm to hot environments, will be the primary avenue for heat loss (Parsons, 2014). Using mean data from the present study at 20°C, the sweat rate required to achieve heat balance would more than double when air speed is 0.7 m s^−1^ compared to at 10.8 m s^−1^ (1.7 vs. 0.75 L h^−1^). Under the same parameters, the required rate of evaporation would be 260% of the maximal achievable at 0.7 m s^−1^ compared to 95% at 10.8 m s^−1^. Although wind speeds achieved in the present study are still slightly below those experience on the road in a TT, practically the difference between 10.8 and 12.5 m s^−1^ is 0.13 L h^−1^ of sweat and an 8% reduction in the required rate of evaporation in order to achieve heat balance. This is reflected in the *T*_gi_ data presented, where there is a clear plateau in *T*_gi_ after the opening 10 km of the TT in both 10 and 20°C, indicating that heat balance has been achieved. The plateau occurs later in the 30°C condition but is likely because of altered pacing and lower mean power, resulting in a reduced metabolic rate, compared to 10 and 20°C. The present data supports the recent study by Valenzuela et al., (2022) showing a reduction in mean maximal power achieved by professional cyclists in temperatures above 25°C (Valenzuela et al., 2022). Interestingly, there was a tendency for female riders to show impaired performance in the 25–30°C range, whereas their male counterparts experienced a performance decline >30°C (Valenzuela et al., 2022). It could be speculated that as average speeds are higher in the male peloton, males experience higher absolute and relative rates of sweating (Mehnert et al., 2002) and lower rates of heat storage (Burse, 1979) and are therefore able to better tolerate extremes of temperature during exercise. This is compounded by the fact that women typically have a smaller body mass, higher body fat mass, smaller body surface area, and a larger surface area‐to‐mass ratio compared with men (Cheuvront & Haymes, 2001; Epstein et al., 2013; Shapiro et al., 1980). Consequently, it is possible that there are both sex and cardiometabolic effects on an individual's ability to tolerate high intensity exercise in the heat, which are likely heavily influenced by air speed, sweat rate and evaporative potential. This explains why in the present cohort a performance impact is evident above 20°C but < 30°C, although it is not possible to pinpoint an exact temperature threshold from these data. However, considering our previous research in a similar cohort (Faulkner et al., 2015, 2019), and that from the professional peloton (Valenzuela et al., 2022), it is reasonable to suggest 20–25°C as a performance limiting temperature threshold, with higher cardiovascular fitness linked to increased thermal tolerance (Périard et al., 2016) and higher likely threshold temperature, reflecting the sex differences reported by (Valenzuela et al., 2022). Therefore, some of the initial conclusions from Galloway and Maughan (1997) should be reconsidered in light of the underestimation of the impact that air speed and evaporative cooling has on the physiological and performance related responses to high ambient temperatures.

### 4.1 Muscle oxygenation

The present data show an inverse relationship exists between power output and SmO_2_, in agreement with others (Jaime et al., 2019; Kirby, Clark, et al., 2021; Poole et al., 1988; Poole & Richardson, 1997). Furthermore, a clear relationship is evident between ∆SmO_2_ and ∆HR, where greater reductions in SmO_2_ result in higher ∆HR, as a function of increased cardiovascular strain (Pèriard et al., 2011). It has been shown that higher ambient temperatures result in a reduction in SmO_2_ for the same external workload (Geng et al., 2023) which also hinders gross economy (Hettinga et al., 2007), likely occurring owing to a redistribution of blood flow in order to maximize heat loss. Together, this suggests that the reduction in power is the consequence, and not the cause, of a reduced ∆SmO_2_ in the 30°C trial in order to maintain the metabolic cost and an acceptable rate of heat production. In support of this view are our data showing that both mean *T*_sk_ and mean *T*_b_ are highest in the 30°C condition, resulting in altered pacing, but not a reduction in SmO_2_, which only occurs at lower temperatures where power output is higher. It has been suggested that a continual decline in SmO_2_ saturation occurs when exercise intensity is above critical power (CP), whereas no change or an increase in SmO_2_ indicates a sustainable exercise intensity below CP (Kirby, Winn, et al., 2021). The present data reflect this, as mean power for 10°C (281 ± 5 W) and 20°C (274 ± 10 W) conditions was in excess of the participants' mean anaerobic threshold (270 ± 15 W) and likely at or just above CP (MacInnis & Gibala, 2017), whereas the altered pacing in 30°C resulted in mean power (255 ± 10 W) being ~15–20 W below anaerobic threshold and CP. When we consider the ∆SmO_2_ at 30°C being 0.4 ± 0.7%, this suggests that the workload sits within the sustainable intensity domain, with pacing alterations occurring independently of O_2_ delivery to the muscle. This is supported by Kirby, Winn, et al. (2021), who demonstrate that a zero percent SmO_2_ slope appears to describe the highest steady‐state metabolic rate, separating sustainable (below CP) from unsustainable (above CP) external work rates (Kirby, Winn, et al., 2021).

## 5 CONCLUSION

These data show that self‐paced cycling time trial performance is impaired in the heat in temperatures above 20°C, which is considerably higher than previously reported in laboratory conditions. When considering other published data derived from both field and laboratory‐based studies (Faulkner et al., 2019; Valenzuela et al., 2022), it is likely that the individual threshold will lie between 20 and 30°C and be dependent on the endurance capacity and sex of the individual athlete. Above these temperatures, athletes, coaches, and practitioners should consider implementing pre‐ and per‐cooling strategies to mitigate the deleterious impact of elevated ambient temperatures on endurance performance.

## AUTHOR CONTRIBUTIONS

S. H. Faulkner, R. James, and KG conceived and designed the experiments. S. H. Faulkner, K. Griggs, E. Martin, N. Wilson, R. James, and P. Jobling contributed to data acquisition, analysis, and interpretation.

S. H. Faulkner, K. Griggs, E. Martin, and N. Wilson prepared the initial draft of this manuscript. All authors contributed to revisions and approved the manuscript prior to final submission and agree to be accountable for all aspects of the work in ensuring that questions related to the accuracy or integrity of any part of the work are appropriately investigated and resolved. All of the listed authors qualify for authorship.

## FUNDING INFORMATION

No funding was received for the completion of this work.

## CONFLICT OF INTEREST STATEMENT

The authors report no potential conflict of interest arising from this work.

## ETHICS STATEMENT

All procedres and methods used within this study were reviewed and approved by the Nottingham Trent University Invasive Ethical Review board prior to commencment of the study.

## Data Availability

The data that support the findings of this study are available from the corresponding author upon reasonable request.

## References

[phy270434-bib-0001] ASHRAE . (1997). Thermal comfort. ASHRAE handbook of fundamentals. (pp. 8.1–8.26).

[phy270434-bib-0002] Borg, G. A. (1982). Psychophysical bases of perceived exertion. Medicine and Science in Sports and Exercise, 14(5), 377–381. 7154893

[phy270434-bib-0003] Burse, R. L. (1979). Sex differences in human thermoregulatory response to heat and cold stress. Human Factors, 21(6), 687. 10.1177/001872087912210606 393617

[phy270434-bib-0004] Buysse, D. J. , Reynolds, C. F. , Monk, T. H. , Berman, S. R. , & Kupfer, D. J. (1989). The Pittsburgh sleep quality index: A new instrument for psychiatric practice and research. Psychiatry Research, 28(2), 193–213. 10.1016/0165-1781(89)90047-4 2748771

[phy270434-bib-0005] Cheuvront, S. N. , & Haymes, E. M. (2001). Thermoregulation and marathon running biological and environmental influences. Sports Medicine, 31(10), 743–762. 11547895 10.2165/00007256-200131100-00004

[phy270434-bib-0007] Epstein, Y. , Yanovich, R. , Moran, D. S. , & Heled, Y. (2013). Physiological employment standards IV: Integration of women in combat units physiological and medical considerations. European Journal of Applied Physiology, 113(11), 2673–2690. 10.1007/s00421-012-2558-7 23238928

[phy270434-bib-0008] Faulkner, S. H. , Broekhuijzen, I. , Raccuglia, M. , Hupperets, M. , Hodder, S. G. , & Havenith, G. (2019). The threshold ambient temperature for the use of precooling to improve cycling time‐trial performance. International Journal of Sports Physiology and Performance, 14(3), 323–330. 10.1123/ijspp.2018-0310 30160552

[phy270434-bib-0009] Faulkner, S. H. , Hupperets, M. , Hodder, S. G. , & Havenith, G. (2015). Conductive and evaporative precooling lowers mean skin temperature and improves time trial performance in the heat. Scandinavian Journal of Medicine and Science in Sports, 25(S1), 183–189. 10.1111/sms.12373 25943669

[phy270434-bib-0010] Flouris, A. D. , & Schlader, Z. J. (2015). Human behavioral thermoregulation during exercise in the heat. Scandinavian Journal of Medicine & Science in Sports, 25(S1), 52–64. 10.1111/sms.12349 25943656

[phy270434-bib-0011] Galloway, S. D. R. , & Maughan, R. J. (1997). Effects of ambient temperature on the capacity to perform prolonged cycle exercise in man. Medicine and Science in Sports and Exercise, 29(9), 1240–1249. 10.1097/00005768-199,709,000-00018 9309637

[phy270434-bib-0012] Geng, Z. , Wang, J. , Cao, G. , Tan, C. , Li, L. , & Qiu, J. (2023). Differential impact of heat and hypoxia on dynamic oxygen uptake and deoxyhemoglobin parameters during incremental exhaustive exercise. Frontiers in Physiology, 14, 1247659. 10.3389/fphys.2023.1247659 38260100 PMC10801013

[phy270434-bib-0013] Havenith, G. (2001). Individualized model of human thermoregulation for the simulation of heat stress response. Journal of Applied Physiology, 90, 1943–1954. 10.1152/jappl.2001.90.5.1943 11299289

[phy270434-bib-0014] Hettinga, F. J. , De Koning, J. J. , de Vrijer, A. , Wüst, R. C. I. , Daanen, H. A. M. , & Foster, C. (2007). The effect of ambient temperature on gross‐efficiency in cycling. European Journal of Applied Physiology, 101(4), 465–471. 10.1007/s00421-007-0519-3 17661069 PMC2039810

[phy270434-bib-0015] Jaime, S. J. , Pratt, C. , Reinschmidt, P. , Kovacs, A. , Porcari, J. P. , & Foster, C. (2019). Muscle oxygenation patterns during a 20‐km time trial with intermediate sprints and recoveries. Medicine & Science in Sports & Exercise, 51(6S), 465. 10.1249/01.mss.0000561896.68399.9e 30365419

[phy270434-bib-0016] Kirby, B. S. , Clark, D. A. , Bradley, E. M. , & Wilkins, B. W. (2021). The balance of muscle oxygen supply and demand reveals critical metabolic rate and predicts time to exhaustion. Journal of Applied Physiology, 130(6), 1915–1927. 10.1152/japplphysiol.00058.2021 33914662

[phy270434-bib-0017] Kirby, B. S. , Winn, B. J. , Wilkins, B. W. , Jones, A. M. , & Kirby, B. (2021). Interaction of exercise bioenergetics with pacing behavior Running Title: Effect of Race Tactics on D′ Balance and Finishing Position 14 15.

[phy270434-bib-0035] Lim, C. S. , Gohel, M. S. , Shepherd, A. C. , & Davies, A. H . (2009). Iliac Artery Compression in Cyclists: Mechanisms, Diagnosis and Treatment. European Journal of Vascular and Endovascular Surgery, 38(2), 180–186. 10.1016/j.ejvs.2009.03.024 19427244

[phy270434-bib-0018] MacInnis, M. J. , & Gibala, M. J. (2017). Physiological adaptations to interval training and the role of exercise intensity. Journal of Physiology, 595(9), 2915–2930. 10.1113/JP273196 27748956 PMC5407969

[phy270434-bib-0019] Maughan, R. J. , Otani, H. , & Watson, P. (2012). Influence of relative humidity on prolonged exercise capacity in a warm environment. European Journal of Applied Physiology, 112(6), 2313–2321. 10.1007/s00421-011-2206-7 22012542

[phy270434-bib-0020] McNair, D. , Maurice, L. , & Droppleman, L. (1971). Manual profile of mood states. Educational and Industrial Testing Service.

[phy270434-bib-0021] Mehnert, P. , Bröde, P. , & Griefahn, B. (2002). Gender‐related difference in sweat loss and its impact on exposure limits to heat stress. International Journal of Industrial Ergonomics, 29(6), 343–351. 10.1016/S0169-8141(02)00073-2

[phy270434-bib-0022] Otani, H. , Kaya, M. , Tamaki, A. , Goto, H. , & Maughan, R. J. (2019). Exposure to high solar radiation reduces self‐regulated exercise intensity in the heat outdoors. Physiology and Behavior, 199, 191–199. 10.1016/j.physbeh.2018.11.029 30471385

[phy270434-bib-0023] Parsons, K. (2014). Human thermal environments: The effects of hot, moderate, and cold environments on human health, comfort, and performance, third edition. In Human thermal environments: The effects of hot, moderate, and cold environments on human health, comfort, and performance, Third Edition. 10.1201/b16750

[phy270434-bib-0006] Pauw, K. , Roelands, B. , de Geus, B. , & Meeusen, R. (2013). Guidelines to classify subject groups in sport‐ science research. International Journal of Sports Physiology and Performance, 8, 111–122. 10.1123/ijspp.8.2.111 23428482

[phy270434-bib-0024] Pèriard, J. D. , Cramer, M. N. , Chapman, P. G. , Caillaud, C. , & Thompson, M. W. (2011). Cardiovascular strain impairs prolonged self‐paced exercise in the heat. Experimental Physiology, 96(2), 134–144. 10.1113/expphysiol.2010.054213 20851861

[phy270434-bib-0025] Périard, J. D. , Travers, G. J. S. , Racinais, S. , & Sawka, M. N. (2016). Cardiovascular adaptations supporting human exercise‐heat acclimation. Autonomic Neuroscience, 196, 52–62. 10.1016/j.autneu.2016.02.002 26905458

[phy270434-bib-0026] Poole, D. C. , & Richardson, R. S. (1997). Determinants of oxygen uptake: Implications for exercise testing. Sports Medicine, 24(5), 308–320. 10.2165/00007256-199,724,050-00003 9368277

[phy270434-bib-0027] Poole, D. C. , Ward, S. A. , Gardner, G. W. , & Whipp, B. J. (1988). Metabolic and respiratory profile of the upper limit for prolonged exercise in man. Ergonomics, 31(9), 1265–1279. 10.1080/00140138808966766 3191904

[phy270434-bib-0028] Schlader, Z. J. , Simmons, S. E. , Stannard, S. R. , & Mündel, T. (2011). Skin temperature as a thermal controller of exercise intensity. European Journal of Applied Physiology, 111(8), 1631–1639. 10.1007/s00421-010-1791-1 21197543

[phy270434-bib-0029] Shapiro, Y. , Pandolf, K. B. , Avellini, B. A. , Pimental, N. A. , & Goldman, R. F. (1980). Physiological responses of men and women to humid and dry heat. Journal of Applied Physiology, 49(1), 1–8. 10.1152/jappl.1980.49.1.1 7399982

[phy270434-bib-0030] St Clair Gibson, A. , Swart, J. , & Tucker, R. (2018). The interaction of psychological and physiological homeostatic drives and role of general control principles in the regulation of physiological systems, exercise and the fatigue process–the integrative governor theory. European Journal of Sport Science, 18(1), 25–36. 10.1080/17461391.2017.1321688 28478704

[phy270434-bib-0031] Tatterson, A. J. , Hahn, A. G. , Martini, D. T. , & Febbraio, M. A. (2000). Effects of heat stress on physiological responses and exercise performance in elite cyclists. Journal of Science and Medicine in Sport, 3(2), 186–193. 10.1016/S1440-2440(00)80080-8 11104310

[phy270434-bib-0032] Tucker, R. , Marle, T. , Lambert, E. V. , & Noakes, T. D. (2006). The rate of heat storage mediates an anticipatory reduction in exercise intensity during cycling at a fixed rating of perceived exertion. The Journal of Physiology, 574(3), 905–915. 10.1113/jphysiol.2005.101733 16497719 PMC1817748

[phy270434-bib-0033] Valenzuela, P. L. , Mateo‐March, M. , Zabala, M. , Muriel, X. , Lucia, A. , Barranco‐Gil, D. , & Pallarés, J. G. (2022). Ambient temperature and field‐based cycling performance: Insights from male and female professional cyclists. International Journal of Sports Physiology and Performance, 17(7), 1025–1029. 10.1123/ijspp.2021-0508 35338106

